# A Review of Patellar Tendinopathy in Athletes Involved in Jumping Sports

**DOI:** 10.7759/cureus.47459

**Published:** 2023-10-22

**Authors:** John Walton, Erik Kozina, Frank Woo, Shaheen Jadidi

**Affiliations:** 1 Family and Community Medicine, McGaw Medical Center of Northwestern University, Chicago, USA; 2 Orthopedics, University of Illinois at Chicago, Chicago, USA; 3 Internal Medicine and Pediatrics, Loyola University Medical Center, Chicago, USA; 4 Sports Medicine, Loyola University Medical Center, Chicago, USA

**Keywords:** young athlete, collegiate athlete, jumper’s knee, sports medicine, patellar tendonitis

## Abstract

This review article discusses the anatomy and histopathology of the patellar tendon, as well as the risk factors and common interventions for patellar tendinopathy (PT) with a view to guide clinicians in treating athletes with patellar tendon pain. PT, or jumper's knee, refers to a chronic injury to the patellar tendon that affects athletes who engage in jumping and explosive movements. The condition is characterized by degeneration and disorganization of the collagen fibers in the tendon, an increase in mucoid ground substance, and fibroblast proliferation. Risk factors for patellar tendinopathy include participation in jumping sports, a greater counter-movement jump height, and training on hard surfaces. Nonoperative treatments for patellar tendinopathy include relative rest, stretching and strengthening exercises, and correction of biomechanical abnormalities. Surgery and other procedures, such as extracorporeal shockwave therapy (ESWT) and injection therapies, may be considered for patients who do not respond to conservative measures.

## Introduction and background

Patellar tendinopathy (PT), or jumper's knee, is often caused by chronic repetitive injury to the patellar tendon. This type of injury can lead to microtrauma in the tendon, causing pain and tenderness in the knee [1]. This condition is most common among athletes who engage in activities that involve a lot of jumping or explosive movements, such as volleyball and basketball. Up to 40% of high-level athletes in these sports may develop PT [2]. This condition can cause athletes to stop participating in sports altogether, with more than 50% of those affected potentially forced to give up their sports due to PT [3]. This review provides an overview of the anatomy and histopathology of the patellar tendon, as well as the risk factors and common interventions for PT, to help guide clinicians in treating athletes with patellar tendon pain.

## Review

Histology and pathophysiology

PT is a condition characterized by chronic injury to the patellar tendon. Histopathology studies have shown that this type of injury can cause several changes in the structure of the tendon. There is often degeneration and disorganization of the collagen fibers in the tendon, as well as disruption of the normal parallel arrangement of fibrils. This can lead to the development of microscopic to macroscopic intrasubstance tendon tears [4].

In addition to these changes in the structure of the tendon, there is also often an increase in the amount of mucoid ground substance in the tendon. This substance is composed of glycosaminoglycans and proteoglycans, which play a role in maintaining the structural integrity of the tendon. The increased presence of this substance may contribute to the thickening of the tendon, which is often seen in PT [5]. Another change that can occur in the tendon in PT is fibroblast proliferation. Fibroblasts are cells responsible for producing collagen and other structural components of the tendon. In response to injury, these cells may proliferate and produce more collagen, which can contribute to the thickening and scarring of the tendon often seen in PT [6].

In addition, neovascularization may occur in the tendon, which involves the development of new blood vessels in the tendon, which can provide a source of nutrients and oxygen to the injured tissue. Neovascularization may also play a role in developing intratendinous calcifications, deposits of calcium that can form in the tendon. These calcifications often have a gritty, toothpaste-like consistency and may contribute to the pain and stiffness often associated with PT [7].

Epidemiology and risk factors

While an excessive jumping motion is often associated with PT, the risk factors contributing to this condition remain somewhat ambiguous. One of the notable contributing factors is participation in sports that involve frequent jumping or explosive movements. A meta-analysis of existing research has emphasized the significance of 'counter-movement jump height' as a potential risk factor for PT [2]. Specifically, counter-movement jump height is a biomechanical metric commonly used to measure the explosive power of the lower limbs. It involves a downward movement immediately followed by an explosive upward jump, which provides a comprehensive representation of an athlete's concentric and eccentric leg power. High counter-movement jump height often suggests stronger yet more forceful tendon loading during sports activities, which could place additional strain on the patellar tendon over time. Therefore, a greater counter-movement jump height could predispose athletes to increased mechanical stress on the patellar tendon, possibly leading to PT. Risk factors requiring more evidence include greater activity volume, higher body weight, decreased ankle dorsiflexion range of motion, and decreased flexibility in the posterior thigh and quadriceps muscles.

Other potential risk factors for PT include training on hard surfaces, recent use of fluoroquinolone antibiotics, and underlying connective tissue diseases such as rheumatoid arthritis and ankylosing spondylitis [8]. These may increase the risk of injury to the patellar tendon and contribute to the development of PT. As such, it is essential for athletes - and others at risk - to be aware of these potential risk factors and take steps to reduce their risk of injury. This may include avoiding high-impact activities or excessive jumping, maintaining a healthy weight, and engaging in regular stretching and flexibility exercises to help maintain good flexibility in the muscles around the knee.

Diagnosis

Diagnosing PT can be challenging, as it is a clinical diagnosis, and routine imaging is neither required nor recommended [3]. In diagnosing PT, a healthcare provider will typically begin by taking a thorough history, including questions about the onset and progression of pain in the knee, as well as any activities or sports that the patient participates in. Common presenting symptoms of PT are pain and tenderness around the patella tendon, pain with jumping or bending and straightening the knee, and swelling. The provider may also ask about any other factors that may be contributing to the development of PT, such as training on hard surfaces, recent use of fluoroquinolone antibiotics, or underlying connective tissue diseases [9].

Next, the provider will perform a physical examination of the patient's knee, which includes evaluating the patient's range of motion, flexibility, and strength. The provider may also test for tenderness at specific points along the patellar tendon, including the origin (inferior pole of the patella), the midportion of the tendon, and the insertion (tibial tubercle). Pain reproducible along the patellar tendon and exacerbated by knee extension against resistance or with maximal stretching of the quadriceps muscles is classically indicative of PT [10].

In addition to these clinical tests, the provider may also order imaging tests to help confirm the diagnosis. Plain film radiographs may show occasional intratendinous calcifications, while MRI or ultrasound (US) may reveal increased signals within the patellar or quadriceps tendon or neovascularization on color Doppler flow [11]. However, the study of choice and relevance of abnormalities remain somewhat controversial, and these tests are not routinely recommended [12]. A diagnosis of PT should not be made based solely on imaging studies, as these can be misleading. Instead, the diagnosis should be made based on the patient's symptoms, physical examination, and response to treatment [13].

Nonoperative treatments

The initial management of PT includes pain and load management. Nonsteroidal anti-inflammatory drugs (NSAIDs) and acetaminophen may be used sparingly for short-term pain management. The use of NSAIDs remains controversial as inflammation plays little-to-no role in the pathogenesis of PT or other tendinopathies [14]. Additionally, NSAIDs may inhibit tendon healing. Inflammation plays a crucial role in the natural healing process of tendons. NSAIDs inhibit tenocyte migration and proliferation and are, therefore, not recommended in the long-term treatment of PT [15]. Rest is essential to prevent further damage and allow the tendon to heal; however, relative rest is preferred over immobilization to prevent muscle or tendon atrophy [16].

Rehabilitation

Physical therapy is the mainstay of conservative treatment for PT. It has been demonstrated to improve strength and flexibility in the knee extensor mechanism, which may help alleviate symptoms and prevent further injury [17]. The most effective exercise modality, however, is still under investigation. Eccentric exercise therapy (EET) is the most commonly used modality by physical therapists, as its use is supported by substantial evidence [18]. Specifically, the quadriceps muscle and patellar tendon are strengthened via exercises emphasizing eccentric contraction of the quadriceps muscle. This promotes the remodeling of tendonous collagen fibers in a slow, gradual process of microscopic tendonous repair via mechanotransduction. However, EET has several limitations. It is pain-provoking for patients, and it is unclear whether it is a beneficial therapy during the competitive season [19].

Kongsgaard et al. compared EET with heavy, slow resistance exercise (HSR) and steroid injections. Their findings suggest that HSR is as effective as EET in improving pain, pathology, and fiber remodeling while providing higher patient satisfaction [20]. Additionally, a randomized controlled trial by Breda et al. found progressive tendon-loading exercises (PTLE) superior to EET at 24 weeks [21]. PTLE is based on progressively increasing tendon loading in a pain-limited fashion via the following four-stage exercise progression: isometric (static), isotonic (dynamic), energy storage (explosive), and sport-specific exercises [22]. 

Bracing and Counterforce Straps

Counterforce straps serve as an adjunct to both the initial management and rehabilitation of PT. These straps are applied around the proximal patella to redistribute stress away from the tendon, providing a mechanical advantage that can alleviate localized pain during activity. The straps may be particularly useful for athletes or individuals engaged in activities that involve frequent jumping or rapid directional changes, offering a supplemental method for managing symptoms and enabling more effective participation in physical therapy [22].

Extracorporeal Shockwave Therapy

Extracorporeal shockwave therapy (ESWT) was proposed as a promising adjunct therapy for PT by van Leeuwen et al., as it is simple to administer, safe, and not pain-provoking [23]. Several theories illustrate the utility of ESWT for the treatment of PT. As described by van der Worp H et al., ESWT can (1) diminish the transmission of pain signals to the brain via hyper-stimulation of the painful area, (2) support tissue regeneration of the tendon, and (3) destroy tissue calcification [24]. 

However, there is conflicting evidence, in support of its use for treating PT. Several randomized controlled trials have concluded that ESWT is superior to either other conservative treatments or sham procedures for other musculoskeletal conditions [25-27]. Others have concluded ESWT to have no difference in effect compared to sham treatments [28,29]. Several systematic reviews and meta-analyses have, unfortunately, still reached conflicting conclusions, as several articles support ESWT while others found no differences between ESWT and conservative treatments [23-25,30]. 

Given the lack of consensus on results, further studies are required before ESWT can be definitively recommended for treating PT. Although ESWT has limited evidence, it is considered a safe procedure that may help some patients. Additionally, ESWT may increase muscular power and strength when combined with exercise training and stretching [31]. As such, clinicians should use their clinical judgment when offering this experimental treatment for PT. 

Injection Therapy

Platelet-rich plasma (PRP): PRP injections allow the clinician to increase the concentration of growth factors locally. It is theorized that increasing the concentration of local growth factors can promote tissue healing and remodeling. As such, it has been proposed as an adjuvant - and promising - treatment in many soft tissue conditions, including PT [32]. 

The evidence supporting PRP in treating PT is conflicting. A prospective cohort study by Gosens et al. compared PRP in patients who had previously failed invasive treatments (ethoxysclerol, cortisone, and/or surgery) with patients who had not received prior treatment. They concluded that PRP was effective in both cohorts but was more effective in the group without previous treatment [33]. A systematic review by Jeong et al. further supported that PRP is an effective treatment option for PT [34]. However, their evidence was poor as most studies in their review were non-RCTs, case studies, and retrospective studies.

PRP can be subdivided into different types, typically based on the concentration of white blood cells present: leukocyte-poor (LP)-PRP and leukocyte-rich (LR)-PRP [35]. There is debate as to whether the presence of leukocytes in PRP is beneficial or detrimental in healing [34,35]. Chen et al. conducted a network meta-analysis that compared several different nonoperative treatments for PT and found LR-PRP to have the most significant effect. They looked at LR-PRP, cortisone injections, ESWT, autologous blood, DN, and topical glyceryl trinitrate (GN). They concluded that LR-PRP led to the greatest improvement in pain and functional outcomes but also noted that findings on treatment effects may have been biased [36]. 

Barman et al. conducted a meta-analysis and found no differences in pain reduction, functional outcomes, and quality of life between PRP and other injections. However, they found PRP to be more effective than ESWT [37]. A recent meta-analysis by Masiello et al. studied the use of PRP in treating various tendinopathies. They found that PRP was more effective than other experimental interventions only in the treatment of carpal tunnel syndrome. They concluded that there were no apparent between-group differences in outcomes in treating lateral epicondylitis, plantar fasciitis, or Achilles, rotator cuff, and patellar tendinopathy [38]. 

Most studies that compared experimental treatments to PRP also included some form of exercise in both control and experimental groups. Additionally, there is a great deal of heterogeneity in outcome measures, injection techniques, and control groups in many of the current RCTs throughout the literature. Further, well-controlled studies are needed, as there is currently insufficient evidence to confirm or deny the effectiveness of PRP in treating PT. In clinical practice, we recommend using other evidence-based modalities before turning to PRP as an adjuvant therapy. 

Corticosteroids: the use of corticosteroid injections (CORT) in treating PT has been well-studied. CORT plays a role in reducing inflammation and pain in the short term; however, CORT use seems to limit long-term healing and functional outcomes and increases the risk of tendon rupture as evidenced by several recent review articles; it is becoming widely accepted that CORT use has little-to-no role in treating PT [39,40].

Hyaluronic acid (HA): HA is naturally found within the extracellular matrix of soft connective tissues and synovial fluid. While traditionally used for the treatment of degenerative joint diseases such as arthritis, its role in soft tissue injuries was further investigated by Khan et al. in a meta-analysis, which concluded that, as a class, HA may be effective in reducing pain and improving function in soft tissue injuries [41]. In an RCT, Kaux et al. found HA to be as effective as PRP in treating PT in the medium term; however, in the long term, HA was not as effective in pain reduction or quadriceps strength [42]. In contrast, Challoumas et al. conducted two network meta-analyses that looked into the management options of tendinopathies and concluded that HA was one of the only treatments that was found promising when combined with exercise [43]. 

Topical Glyceryl Trinitrate 

Topical glyceryl trinitrate (GTN) was proposed as an adjuvant treatment for rotator cuff tendinopathy in 1996 [44]. Over two decades later, however, there is still only a limited amount of evidence in support of its use in PT. It is theorized that the mechanism by which GTN alleviates tendinopathies is by increasing the amount of nitric oxide, which leads to vasodilation and, eventually, stimulates collagen production [45]. A meta-analysis of eight RCTs by Saltychev et al. looked into the use of GTN in treating several tendinopathies and found no difference between GTN and placebo [46]. In contrast, Challoumas et al., in their network meta-analysis, listed GTN, along with HA, as one of the only promising interventions in treating PT [43]. However, there was only one RCT of GTN in their analysis. As such, there is still limited data supporting the use of GTN in treating PT. Furthermore, GTN use is associated with the well-known side effect of headaches. 

Percutaneous Needle Tenotomy

Chronic PT can develop when conservative and above-mentioned management options for PT fail to improve patients' symptoms. Percutaneous needle tenotomy (PNT) is a promising outpatient procedure that may help treat chronic PT. PNT is used to cut and debride out damaged tendinopathic tissue while preserving healthy surrounding tissue. This can help promote pain relief and functional recovery when other treatment modalities options have failed. Maffulli et al. analyzed percutaneous longitudinal internal tenotomy (PLIT) use in a study involving 38 athletes with PT and concluded that it could be the first-line, minimally invasive operative intervention in the treatment of chronic mid-substance PT when conservative management failed [47]. PLIT was not found to be effective in patients with insertional tendinopathy. Although PNT has the potential to be an alternative treatment option before moving to major operative intervention, as stated above, more research is needed to understand its efficacy in treating PT. 

Operative treatment

Non-surgical treatment is effective in managing PT in up to 90% of cases, based on criteria that include pain reduction, functional improvement, and the ability to return to sports [48]. However, in rare cases where patients do not respond to conservative treatments, including exercise therapy, surgical intervention may be considered. Surgery is generally advised only after six months of unsuccessful nonoperative treatment [49].

There are two prevalent surgical techniques for treating PT: open and arthroscopic surgery. Both techniques focus on the excision of pathological tissue from the patellar tendon. According to existing research, neither technique has proven to be statistically superior to the other in terms of overall outcomes, as measured by postoperative pain levels, functional improvement, and return to sports activities. Specifically, success rates are 87% for open surgery and 91% for arthroscopic surgery. However, the average rate of return to sports is quicker with arthroscopic surgery (82%) compared to open surgery (78%) [43,50].

Given these statistics, arthroscopic surgery is often the preferred technique, provided the surgeon has adequate experience with the method. Moreover, certain surgical approaches, such as the removal of the inferior pole of the patella, have been described to be potentially beneficial for treating chronic cases of PT. This technique aims to eliminate the source of mechanical irritation contributing to tendinopathy [49,50].

## Conclusions

PT, also known as jumper's knee, is a condition that can affect athletes who engage in activities involving jumping and explosive movements. This condition is caused by chronic repetitive injury to the patellar tendon, which leads to changes in the structure of the tendon, such as degeneration and disorganization of the collagen fibers and an increase in mucoid ground substance. The risk factors for PT include participation in jumping sports, a greater counter-movement jump height, and training on hard surfaces. Nonoperative treatments, such as relative rest, stretching and strengthening exercises, and correction of biomechanical abnormalities, can effectively manage the condition. However, in cases where these treatments are not effective, surgery or other procedures may be necessary.
